# Special Issue: “Environmentally Friendly Polymeric Blends from Renewable Sources”

**DOI:** 10.3390/ma14174858

**Published:** 2021-08-26

**Authors:** Barbara Gawdzik, Olena Sevastyanova

**Affiliations:** 1 Department of Polymer Chemistry, Maria Curie-Sklodowska University, 20-614 Lublin, Poland; 2 Wallenberg Wood Science Center, Department of Fibre and Polymer Technology, KTH-The Royal Institute of Technology, Teknikringen 56-58, 10044 Stockholm, Sweden; olena@kth.se

## 1. Introduction

The aim of this Special Issue is to highlight the progress in the manufacturing, characterization, and applications of environmentally friendly polymeric blends from renewable resources.

Materials from renewable resources have attracted increasing attention in past decades as a result of environmental concerns and due to the depletion of petroleum resources. Polymeric materials from renewable sources have a long history. They were already used in ancient times and later accompanied man along with the development of civilization. Currently, they are widespread in many areas of life and used, for example, in packaging, and in the automotive and pharmaceutical industries.

Polymers from renewable resources are generally classified into three groups: (i) natural polymers, such as cellulose, starch and proteins; (ii) synthetic polymers from natural monomers, such as poly(lactic acid), and (iii) polymers from microbial processes, such as poly(hydroxybutyrate). The emergence of new methods and analytical tools provides a new level of understanding of the structure–property relationship of natural polymers and allows the development of materials for new applications.

One of the attractive properties of the natural polymers and synthetic polymers produced from natural monomers is their inherited biodegradability. On the other hand, this is related to their moisture sensitivity, which limits their application. Other important limitations of most polymers from renewable resources are their lower softening temperature and mechanical strength. These and many other properties of polymers can be modified and improved through the blending of two or more compounds, for example two or more polymers, polymers and fibers, polymers and nanoparticles etc.

A blending approach, which may result in both polymer blends or composites, is an effective way to achieve a desirable combination of properties that are often absent in the individual components. Polymer blends and composites are useful as they can be produced from low-cost raw materials, including industrial waste products, without sacrificing their desired properties; they can also be used to prepare high-performance compounds for broader applications due to biodegradability and reusability of the end products.

The final properties can be modified by changing the relative concentration and kind of monomeric units used in the synthesis or by varying the proportion of homopolymers and various additives in a blend composition. Development of effective methods of manufacturing products from blends of renewable polymers and environmental friendly synthetic polymers in a controlled way is the challenge of our time.

Accordingly, in this Special Issue of Materials, which is aimed at recognizing the current state of knowledge and development in the use of environmentally friendly polymeric blends from renewable sources, the following aspects were investigated:

- synthesis of composites based on natural fillers;
- chemical modification of polymers or fillers in order to improve interfacial interactions;
- potential applications of the biobased materials.

## 2. Short Description of the Articles Presented in This Special Issue

In this Special Issue, 13 original articles have been published on various topics, including the preparation, characterization, and some examples of uses of polymeric materials from renewable resources. This issue includes five articles on polyurethanes blended with various types of additives [1,2,3,4,5], of which two papers discusses the polyurethane systems partly prepared from naturally-derived components, such as lignin [5] or naturally-derived polyols [4]. Four papers discuss the preparation of synthetic unsaturated polyester resins reinforced with natural fillers [6,7,8,9], and two other papers are focused on the modification of polyolefins (PE,PP) to improve their strength properties [10,11]. One work is concerned with the preparation of biochar and bio-oil from biomass, where the potential uses of both types of products are discussed [12]; the other paper considers the modification of cotton linter for use as efficient adsorbent for the phosphate.

Most of above papers pay particular attention to the role of eco-friendly fillers, i.e., microcrystalline cellulose, wood flower, lignin, rice straw nanofibers, lavender residues, sugar beet pulp, barley fibers, hemp shives, walnut shells, and curauá fibers in the improvement of various properties in the final materials.

In the papers of Członka, Strąkowska et al., problems of polyurethane composites with different biofillers were discussed.

In the paper “Polyurethane Hybrid Composites Reinforced with Lavender Residue Functionalized with Kaolinite and Hydroxyapatite” [1], blends were obtained with lavender fillers functionalized with kaolinite and hydroxyapatite. The obtained materials were characterized with enhanced mechanical, thermal, and performance properties.

In the paper “Rigid Polyurethane Foams Reinforced with POSS-Impregnated Sugar Beet Pulp Filler” [2], blends were reinforced with sugar beet pulp impregnated with aminopropyl isobutyl-polyhedral oligomeric silsesquioxanes. The results showed that the greatest improvement in physicomechanical properties was observed at a lower concentration of filler.

In the paper “Impact of Hemp Shives Impregnated with Selected Plant Oils on Mechanical, Thermal, and Insulating Properties of Polyurethane Composite Foams” [3], materials reinforced with hemp shives fillers were synthesized with three different types of fillers: nontreated filler, filler impregnated with sunflower oil, and filler impregnated with tung oil. The incorporation of impregnated biofillers resulted in the improvement of thermal stability and flame retardancy of foams.

In the paper “Rigid Polyurethane Foams Based on Bio-Polyol and Additionally Reinforced with Silanized and Acetylated Walnut Shells for the Synthesis of Environmentally Friendly Insulating” [4], composites produced from walnut shell-derived polyol (20 wt. %) were also reinforced with nontreated, acetylated, and silanized biofiller from walnut shells. The composites with the addition of acetylated and silanized filler exhibited a more uniform structure than foams with the addition of nontreated filler.

In the paper of Gonçalves, Rudnitskaya, Sales, Costa, Evtuguin (“Nanocomposite Polymeric Materials Based on Eucalyptus Lignoboost^®^ Kraft Lignin for Liquid Sensing Applications” [5]) polyurethane–lignin copolymer blended with carbon multilayer nanotubes was used in all-solid-state potentiometric chemical sensors. The interaction between carbon nanotubes and lignin molecules in the polymer enhanced its electrical conductivity.

The papers of Delgado-Aguilar et al. concerned eco-friendly blends derived from polyolefines.

The paper of Delgado-Aguilar, Tarrés, Marques, Espinach, Julián, Mutjé, and Vilaseca (“Explorative Study on the Use of Curauá Reinforced Polypropylene Composites for the Automotive Industry” [10]) studied the properties of composites reinforced with natural fibers (curauá). The results showed that the tensile properties were similar to uncoupled glass fiber-based composites.

In the paper of Serra-Parareda, Tarrés, Delgado-Aguilar, Espinach, Mutjé, and Vilaseca (“Biobased Composites from Biobased-Polyethylene and Barley Thermomechanical Fibers: Micromechanics of Composites” [11]), barley fibers were used as reinforcement for a polyethylene blend. Grafted polyethylene was used as a coupling agent to increase in interfacial adhesion.

Another two papers were devoted to synthetic resin reinforced with various bio-based fillers.

In the paper of Chabros, Gawdzik, Podkościelna, Goliszek, and Pączkowski (“Composites of Unsaturated Polyester Resins with Microcrystalline Cellulose and Its Derivatives” [7]), modified and unmodified microcrystalline cellulose were used as a filler for unsaturated polyester resin composites. The results showed that especially for modified cellulose, the properties of composites were similar to those with the addition of nanocellulose, but incorporating nanocellulose into the resin was much more difficult.

In the paper of Pączkowski, Puszka, Miazga-Karska, Ginalska, and Gawdzik (“Synthesis, Characterization and Testing of Antimicrobial Activity of Composites of Unsaturated Polyester Resins with Wood Flour and Silver Nanoparticles” [6]) the properties of the wood–resin composites were studied. For composites with wood flour, deterioration of mechanical properties was observed. Antimicrobial activity tests showed that bacterial strains can colonize the surface of the cross-linked unsaturated polyester resin. Composites with the addition of wood flour are a good surface for bacteria colonization and their elimination may occur when nanosilver is added to the composite.

In the paper of Alcántara, González, Pareta, and Vilaseca (“Biocomposites from Rice Straw Nanofibers: Morphology, Thermal and Mechanical Properties” [8]), cellulose nanofibers were extracted from rice straw and reinforced the poly(vinyl alcohol) matrix. The results show that Young’s modulus of the polymer increased by three and a half times after adding nanofiller.

In the paper of Wang, Liu, Duan, Xu, Zhang, She, and Zheng (“The Eco-Friendly Biochar and Valuable Bio-Oil from Caragana korshinskii: Pyrolysis Preparation, Characterization, and Adsorption Applications” [12]) biochar and bio-oil were obtained from caragana biomass carbonization in different pyrolysis conditions and the adsorption and pharmaceutical properties of these products were discussed.

The papers of Du at al. and Goliszek at al. concern synthesis of sorbents based on natural components.

In the paper of Du, Dong, Lin, Yang, and Zhao (“Radiation Synthesis of Pentaethylene Hexamine Functionalized Cotton Linter for Effective Removal of Phosphate: Batch and Dynamic Flow Mode Studies” [13]), quaternized cotton linter fibers were prepared by electron beam preirradiation grafting technology. The results showed that the modified cotton linter fibers can be used as sorbents for phosphate removal.

The paper of Goliszek, Podkościelna, Sevastyanova, Gawdzik, and Chabros (“The Influence of Lignin Diversity on the Structural and Thermal Properties of Polymeric Microspheres Derived from Lignin, Styrene, and/or Divinylbenzene” [9]) investigates the impact of lignin influence on the properties of the porous biopolymeric microspheres. It was found that the materials have a high thermal resistance and the incorporation of methacrylated lignin into the microspheres resulted in an increase of specific surface area and porosity.

## 3. Conclusions

The usage of polymer blends and composites, both prepared by blending, are two strategies used in the “green” requirements of many industries. Green chemistry strategies are mainly accomplished by reducing waste production, reducing raw material usage, reducing nonrenewable energy sources and overall energy demand, reducing risks, hazards, and costs. In this Special Issue, the great potential of agricultural and wood residuals, as they are or chemically modified, for the improvement of broad range of polymeric materials is clearly demonstrated. Cheap and abundant resources such as wood, rice straw, hemp, walnut shells, and other types of biomass and their constituents can improve the strength, thermal and some specific properties of final polymeric materials that have a great potential to be used in automotive, construction, and pharmaceutical and environmental protection industries.

## Data Availability

Not applicable.
